# Acoustic and Genetic Data Can Reduce Uncertainty Regarding Populations of Migratory Tree-Roosting Bats Impacted by Wind Energy

**DOI:** 10.3390/ani12010081

**Published:** 2021-12-30

**Authors:** Amanda M. Hale, Cris D. Hein, Bethany R. Straw

**Affiliations:** 1 Department of Biology, Texas Christian University, Fort Worth, TX 76129, USA; 2 National Renewable Energy Laboratory, Arvada, CO 80007, USA; Cris.Hein@nrel.gov; 3 Fort Collins Science Center, U. S. Geological Survey, Fort Collins, CO 80526, USA; bstraw@usgs.gov

**Keywords:** acoustic monitoring, hoary bat, migratory bats, NABat, population genetics, population genomics, renewable energy, tree bats, wind turbine

## Abstract

**Simple Summary:**

Although wind energy provides valuable environmental and economic benefits, it has unintended negative consequences for wildlife, particularly birds and bats that collide with the rotating blades. In North America, for example, there are increasing concerns that wind turbines threaten the persistence of populations of migratory tree-roosting bats, such as the hoary bat (*Lasiurus cinereus*). Because it is not possible to monitor population sizes for these solitary and cryptic species using traditional techniques, we must rely on other methods, such as acoustic and genetic monitoring, to provide information regarding population status and trends. Using these approaches, we can build a weight of evidence to assess whether mortality associated with wind turbines is sustainable and determine if and when mitigation measures should be implemented to reduce wind turbine mortality. To this end, we recommend that acoustic data be collected using the North American Bat Monitoring Program (NABat) protocols, and that genetic diversity be monitored at repeated time intervals to document species trends. There are no short-term measures to resolve population-level questions for migratory tree-roosting bats. Nonetheless, we discuss opportunities for relatively short-term investments that will lead to long-term success in reducing uncertainty for these species.

**Abstract:**

Wind turbine-related mortality may pose a population-level threat for migratory tree-roosting bats, such as the hoary bat (*Lasiurus cinereus*) in North America. These species are dispersed within their range, making it impractical to estimate census populations size using traditional survey methods. Nonetheless, understanding population size and trends is essential for evaluating and mitigating risk from wind turbine mortality. Using various sampling techniques, including systematic acoustic sampling and genetic analyses, we argue that building a weight of evidence regarding bat population status and trends is possible to (1) assess the sustainability of mortality associated with wind turbines; (2) determine the level of mitigation required; and (3) evaluate the effectiveness of mitigation measures to ensure population viability for these species. Long-term, systematic data collection remains the most viable option for reducing uncertainty regarding population trends for migratory tree-roosting bats. We recommend collecting acoustic data using the statistically robust North American Bat Monitoring Program (NABat) protocols and that genetic diversity is monitored at repeated time intervals to show species trends. There are no short-term actions to resolve these population-level questions; however, we discuss opportunities for relatively short-term investments that will lead to long-term success in reducing uncertainty.

## 1. Introduction

Understanding the structure and dynamics of a population can assist in evaluating the scope and severity of a stressor, and the efficacy of mitigation measures. For most species of bats, population data are limited or nonexistent, making it difficult to assess natural and human-induced stressors [1,2,3]. Despite this uncertainty, there is concern regarding bat populations around the world because bats are long-lived and have low reproductive rates; thus, requiring high adult survivorship to avoid population declines [4,5]. This combination of life history traits prohibits populations from recovering quickly from broadscale impacts that may put populations at risk [6,7].

**Figure 1:**
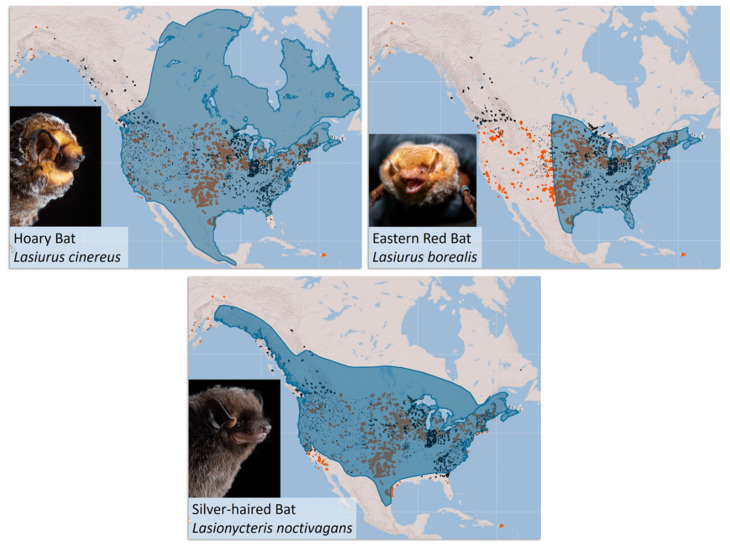
Wind energy deployment in the United States, depicted in orange [24], and NABat grid cells that contain manually vetted acoustic bat monitoring data for each species, represented in black, are overlaid with the species range [25]. Note: these are not presence/absence maps. Hoary bat and silver-haired bat photos reproduced with permission from Jose Martinez-Fonseca, Northern Arizona University; eastern red bat photo reproduced with permission from Will Seiter, Copperhead Environmental Consulting, Inc. Maps are adapted from the North American Bat Monitoring Program Partner Portal [26].

The global expansion of wind energy development represents a potential threat to certain bat populations. In North America, wind energy development primarily impacts migratory tree-roosting species, including hoary bats (*Lasiurus cinereus*), eastern red bats (*Lasiurus borealis*), and silver-haired bats (*Lasionycteris noctivagans*). Note: we elected to use the traditional taxonomy recognized by the taxonomic nomenclature authority and database (www.batnames.org (accessed on 11 November 2021)); [8]); see [9,10] for suggested taxonomic revisions. These bat species have expansive ranges that overlap extensively with existing wind energy development (Figure 1). Based on fatality data from 1999 to 2011, Arnett and Baerwald [11] estimated that hundreds of thousands of bats were killed annually by wind turbines across the United States and Canada. Nearly 78% of the reported fatalities were from hoary bats (38%), eastern red bats (22%), and silver-haired bats (18%) [11]. Since the time of the analysis, installed capacity has increased from ~7.8 and 47 GW to 13 and 125 GW in Canada and the United States, respectively [12,13]. Given the recent and anticipated future growth of wind energy in the United States alone [14,15], the wind industry, regulatory agencies, and conservation organizations are increasingly concerned about the potential population-level impact of wind turbine-related mortality on these species.

Placing wind turbine-related bat mortality into context has been challenging because estimating census population size (Nc) using traditional methods is impractical for migratory tree-roosting bats. These species roost individually, are dispersed across the landscape, and migrate over long distances [16,17]. In lieu of empirical population data, Frick et al. [18] used expert elicitation to inform models regarding the impact of wind energy on hoary bats. Holding installed capacity and fatality rates constant from 2014 data, and assuming a starting population size of 2.25 million bats and a population growth rate of λ = 1.01, the model indicated a 90% population decline in hoary bats by 2050. Friedenberg and Frick [19] updated the models to incorporate future wind development and the implementation of mortality minimization measures. Under the lowest-risk scenario, which included the maximum growth rate (λ = 1.18), the lowest projected development scenario (165 GW), and the median starting population (2.25 million bats), hoary bats are predicted to experience a 50% decline by 2028. The authors recognize the uncertainty in these models, particularly for the starting population size. In addition, the lower bound for new estimates for wind energy development by 2035 may exceed 500 GW [20], a noticeable increase from future development scenarios used in the models.

The lack of available demographic data for many bat species, including hoary bats, emphasizes the importance of collecting data to determine vital rates; however, these studies are labor intensive and may require ≥5 years of data from capturing, marking, and tracking individuals [21]. Although these data are necessary to estimate population growth rates and develop models relating the impacts of a stressor on a population [18,19], collecting such data remains logistically and financially challenging. Nonetheless, integrated population models and modeling software such as the BatTool [22], an R package that allows users to evaluate population level effects of different scenarios, could provide opportunities to integrate data from the North American Bat Monitoring Program (NABat [23]) with general demographic models to improve inferences. Furthermore, the models from Frick et al. [18] and Friedenberg and Frick [19] may be more sensitive to population size than growth rate, indicating the importance of assessing the current population size of hoary bats to assess trends over time.

There are no practical, short-term measures to determine Nc for migratory tree-roosting bats, but additional data can be collected to reduce uncertainty and assist in determining population trends [27,28]. The combination of multiple approaches, such as systematic acoustic sampling and genomic analyses, can build a weight of evidence to inform future modeling efforts. Here, we synthesize available research related to bat populations, uncertainty around existing estimates, methods for evaluating population-level data, and recommendations for short- and long-term studies to reduce uncertainty on the population status and trends of migratory tree-roosting bats. The synthesis and recommendations we provide build on the ideas presented by Hein et al. [28]. Although the focus is on wind energy development and migratory tree-roosting bats in North America, the approaches presented are broadly applicable and are being implemented by several government agencies, industries, academia, and nongovernmental organizations.

## 2. Population Monitoring Using Acoustic Surveys

In 2012, a workshop on bat population monitoring and modeling was convened to design statistically robust and logistically feasible methods for monitoring changes in bat populations [29]. At the workshop, five sampling methods were identified for different species, such as colony counts for subterranean-roosting species. For solitary tree-roosting species, acoustic surveys were determined to be the most appropriate tool for monitoring based on the relatively low cost and the ability to simultaneously survey for multiple species. Workshop participants acknowledged that significant coordination was needed to implement the survey design and sampling methods they identified across Canada, the United States, and Mexico. This spurred the development of NABat [30], a multiagency, multinational effort to address the lack of standardized monitoring for bats [23]. NABat is a collaborative continentwide program designed to monitor bat distribution and abundance from local to range-wide scales [31]. To assess trends in abundance and distribution across spatial and organization scales, NABat uses a hierarchical master sample survey design, integrated data analysis, dynamic data curation, regional monitoring hubs, and knowledge delivery through web-based infrastructure [31].

For acoustic sampling, NABat uses a systematic grid-based framework of 100 km^2^ cells and probability-based sampling [32,33,34,35,36]. A generalized random-tessellation stratified survey [37] generates spatially balanced and randomized priority cells [31]. Because repeat samples are necessary to assess trends, priority cells should be resampled for several years. The number of sampling locations and years required to determine population trends will vary based on several factors, including the commonality of the species. For example, to detect a slight population decline in a rare species will require more sites and years of sampling than a steep decline in a common species. Statistical power analysis can guide studies assessing population change [38]. Based on simulations, for example, Roche et al. [39] reported that it should take 8 years to detect a 50% decline for the common pipistrelle (*Pipistrellus pipistrellus*), whereas it would take approximately 15 years to detect a 25% decline in this species.

Repeated annual surveys at priority grid cells permits the extension of the model to unsampled areas. As a result, changes in occupancy dynamics, or the probability of a site being unoccupied in one year given it was occupied the previous year, can be estimated [40]. Rodhouse et al. [41] assessed occupancy between 2003 and 2010 and again from 2016 to 2018 in Oregon and Washington, U.S.A. Using a multi-season occupancy model, they reported a summertime decline for hoary bats, but did not detect a decline for little brown bats (*Myotis lucifugus*). The inference of the study was limited as it was regionally focused and included only 5% of the continental United States; thus, conclusions about the population status of the two species at larger spatial scales could not be determined. Rodhouse et al. [41] indicated that a range-wide replication of the study would be warranted. The authors concluded that empirically informed Bayesian modeling, driven by large monitoring data sets from a robust survey design and accumulated over time, such as those obtained from NABat, would provide a powerful foundation for building an adaptive, evidence-based conservation information system.

**Figure 2:**
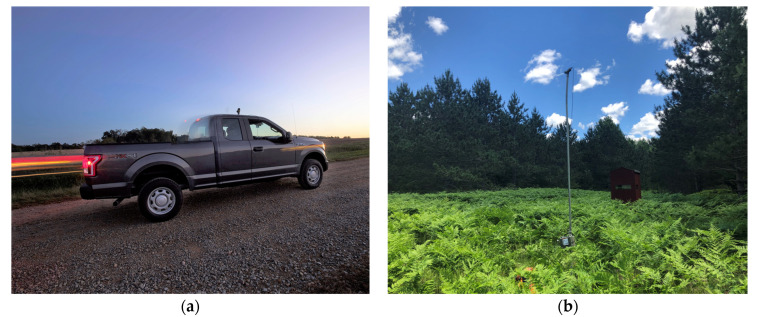
Image of a mobile acoustic transect (**a**) being conducted in North Carolina, United States., reproduced with permission from Melissa McGaw, North Carolina Wildlife Resource Commission. There are currently more than 1.142 million mobile acoustic records in the NABat database [25]. Image of a stationary acoustic deployment (**b**) in Northern Michigan, United States, reproduced with permission from Crystall Birdsall. There are currently more than 62.285 million stationary acoustic records in the NABat database [25].

For NABat, acoustic surveys can be either mobile or stationary. For stationary surveys, as were used in Rodhouse et al. [41], each recorder is deployed at a location for four nights. The deployment includes two to four detectors per 10 km × 10 km grid cell. The recommendation is to position one detector in each of the four 5 km × 5 km quadrants to minimize spatial autocorrelation. The response variable is detection/non-detection, which is used to measure trends in occupancy and habitat use. Stationary surveys are useful if roads are limited, and they can eliminate road bias; however, stationary surveys cannot provide reliable estimates of abundance or density because the number of passes detected do not correspond to the number of bats present. In contrast, mobile transects sample spatial, temporal, and interspecific variation by sampling diverse habitats over large areas [42] (Figure 2). They are most applicable for easily accessible roads that can be safely driven at a speed of 32 km/h (20 mph). Mobile transects should be between 25 and 48 km in length, occur for 1 to 2 h, and take place on two nights within the same week [23].

Mobile transects assume each bat pass represents a unique individual because the vehicle is presumably traveling faster than most bats are flying. Since most bats in North America fly between 9 and 32 km/h, mobile transects are unlikely to encounter the same bat twice [43,44,45,46,47]. The response variables for mobile transects are detection/non-detection for each species and the number of bat passes per species along the transect; thus, an index of relative abundance can be derived from the number of passes [39] to estimate the superpopulation or the total number of individuals estimated to use each grid cell, summed across the modeled range during the survey period. Using analytical methods documented in Doser et al. [48] and Kéry and Royle [49], it is possible to estimate absolute abundance at the grid cell, but because individuals can and are expected to use more than one cell, the estimate is not representative of census population size.

Each survey type, stationary vs. mobile, has advantages and biases. Neece et al. [50] reported significantly higher probabilities of detection at stationary points. Similarly, Whitby et al. [51] noted that mobile transects detected all 12 bat species known to occur in southern Illinois, but it took more sampling events than stationary detectors. On the other hand, however, this study also noted that mobile transects required less effort. In contrast, detections of bats in Texas were greater along mobile transects [52]. Neece et al. [50] suggested using a combination of both approaches within each priority cell. The use of multiple data types in these analyses both provides multiple lines of evidence for inference and increases the sample size to increase confidence and reduce uncertainty.

## 3. Population Monitoring Using Genetic Approaches

Although genetic data cannot provide direct estimates of census population sizes, they are useful for monitoring population status and trends, informing geographic ranges and seasonal movements, and detecting cryptic speciation (i.e., species considered morphologically indistinguishable but genetically distinct and that do not interbreed). These aforementioned aspects of bat biology all require additional investigation and are necessary to properly plan and implement conservation strategies. By combining recent advances in the tools and analytical approaches to monitor genetic variation in wildlife populations with the abundance of bat carcasses that are recovered at certain times of the year in distinct geographic locations across the United States (i.e., during postconstruction fatality surveys at wind energy facilities), these data have the potential to be especially informative. In addition, the ability to monitor for changes in genetic diversity over time could provide unique insights that reflect actual fluctuations in census population size. Although it is not possible to isolate changes in genetic diversity due to a single stressor, the scope and severity of these cumulative changes can guide management and mitigation decisions that are necessary for the conservation of migratory tree-roosting bats.

Genetic variation in a population is essential for adaptation to environmental change, and it is well established that loss of genetic diversity impedes the long-term survival of species [53]. When large populations are reduced in size, genetic diversity declines and natural selection acts less effectively on both advantageous and deleterious alleles. Smaller population sizes also increase inbreeding, thereby exposing deleterious recessive variants that reduce mean population fitness [53,54]. Characterizing the level of genetic diversity within wild populations and its distribution among geographically distinct populations is therefore critical for conserving the integrity and sustainability of species. From a practical perspective, insights from molecular genetic studies have directly influenced the design and implementation of conservation management strategies in wild populations (e.g., [55]).

Population genetic analyses also can detect population bottlenecks, estimate historical and current effective population sizes, and identify evolutionarily unique subpopulations [53]. The effective population size (Ne) is the size of an abstract population with random pairing of gametes and discrete generations that would experience the same amount of genetic drift, or loss of genetic variation due to chance, as the observed population of interest. On average, Ne is 11% (range: 5–80%) of Nc across unmanaged animal populations because anything that increases the variance among individuals in reproductive success (e.g., nonrandom mating) will reduce Ne (reviewed in [56]). Ne is particularly sensitive to variance in family size, an imbalance between males and females, and population size fluctuations [56]. The loss of genetic variation to drift (i.e., because of a severe bottleneck), results in the slow accumulation of new genetic variation, at the underlying mutation rate, causing estimates of Ne to remain low because Ne is most sensitive to the smallest Nc over time. When large populations decline, genetic diversity decreases in predictable ways; therefore, even in the absence of estimates for Nc, continuous genetic monitoring can reveal changes in Ne indicating whether the population is increasing or decreasing [57] and provide direct implications for the conservation and management of species. For example, genetic monitoring of an endangered population of Atlantic salmon (*Salmo salar*) from Maine revealed significant decreases in Ne and several measures of genetic diversity from 1963 to 2001 [58]. These results, in conjunction with a reduction in adult salmon spawning upriver, suggests that existing restoration efforts may be hindered by this historical loss of diversity [58].

Genetic diversity and population structure of migratory tree-roosting bats likely affect resilience to sustained mortality from wind turbines and the likelihood of population persistence. Species with smaller Ne, for example, have lower genetic diversity which may limit their evolutionary potential to respond to selection and avoid inbreeding in the future [53]. Although migratory tree-roosting bats may not be impacted by inbreeding in the short term, it is important to monitor Ne and other measures of genetic diversity to understand if these species are currently losing genetic variation that could influence future adaptation.

Several tools are available to characterize and monitor genetic variation in populations, and recent review papers illustrate how these data can be applied to the conservation of natural populations (e.g., [53,59,60,61]). A recent innovation in detecting genetic variation in natural populations has been the high-throughput application of single-nucleotide polymorphisms, or SNPs [62]. SNP variation in aggregate is abundant in the genome and more tractable to model than microsatellite variation. SNPs also provide improved power to estimate genetic diversity, gene flow, Ne, and other population demographic parameters important for conservation [63,64]. Recently, SNPs have been used to characterize genetic variation in natural populations [65]. The advent of next-generation sequencing methods—such as restriction site-associated DNA sequencing (RADseq), has made it possible to study hundreds or thousands of SNPs across the genome or even low-coverage whole genome sequencing [66]. Genomic studies highlight the importance of recent and even ancient interspecific hybridization, empower greater delimitation of cryptic species, evolutionary significant units, and subspecies [67], and will presumably change how populations are studied and managed [68].

### Population Genetic Studies of Migratory Tree-Roosting Bats

Recent studies using population genetic approaches have elucidated patterns of genetic variation, identified potential barriers to gene flow, and estimated Ne for three migratory tree-roosting bats. For the eastern red bat, several studies have similarly concluded that Ne ranges from hundreds of thousands to millions of individuals, with no evidence of barriers to gene flow among groups of samples [68,69,70,71]. A recent study by Ammerman et al. [72] reported that 71% of parent/offspring groups in eastern red bats consisted of half siblings, which is to date, the highest level of multiple paternity documented for any species of bat. Multiple paternity can increase Ne if it reduces variance in male reproductive success and may be an adaptation to increase genetic variation and litter size [73,74,75]. These attributes may make eastern red bats somewhat more resilient to the genetic consequences of population declines compared to hoary bats. Similar to eastern red bats, no evidence of population genetic structure has been detected in hoary bats [68,70,71], but the estimates of Ne were smaller, ranging from several thousands to hundreds of thousands of individuals, suggestive of either a stable or declining population [70]. Using next-generation sequencing (with thousands of loci), Sovic et al. [71] found no evidence for population structure in the silver-haired bat. For these three migratory tree-roosting bats, they concluded that the silver-haired bat had the smallest estimated current Ne (155,364 to 231,689 bats), followed by the hoary bat (730,470 to 884,046 bats), and then the eastern red bat (1,241,280 to 1,692,254 bats) [71].

It is important to note that the high levels of gene flow and population connectivity observed in these North American bats are based on studies encompassing only a portion of the species’ geographic range. It remains unclear whether these high levels of gene flow and population connectivity exist across their entire geographic ranges. Given the migratory life history of these species, future research should integrate data from the entire range because high levels of mortality in one region may have far-reaching implications. Although no strong genetic evidence of population declines in these species exists, data from other studies suggest their populations may be declining. Korstian et al. [68] cautioned that genetic monitoring of migratory tree-roosting bats, specifically for detecting population declines caused by wind turbines, may be impractical using a limited number of genetic loci given the large effective population sizes and high levels of gene flow in these species. Instead, they suggested focusing on range-wide population genetic studies, developing genomic resources for these species, and obtaining better estimates of mutation rates to better estimate historical and current effective population sizes.

Cornman et al. [76], following up on some of the recommendations from Korstian et al. [68], developed more genomics resources to address sources of statistical bias associated with anonymous markers and implemented a more balanced design than was possible previously to evaluate population trends. Using RADseq techniques, they examined genetic variation in two cohorts of hoary bats, separated by ~7–9 years, collected from wind energy facilities in Indiana, U.S.A. They investigated temporal trends in contemporary Nb, an estimator of Ne that reflects the breeding population size [77] with 95% confidence intervals for each cohort [78]. The point estimates of Nb were not stable across a range of SNP- and sample-filtering strategies; Nb was higher in the 2017–2018 cohort than the 2009–2010 cohort when the full dataset was analyzed, but there was no difference when outliers were removed [76]. Thus, although the cohorts were likely separated by more than the generation time of hoary bats, sampling noise, overlapping generations, and the influence of past Ne can weaken temporal signals, and it was not clear if Nb was increasing, decreasing, or remained stable during this time period. Further, differences in conditions or storage of the samples between the two cohorts affected genotype distributions in a manner consistent with DNA degradation artifacts, adding to the difficulty of making temporal comparisons. Nonetheless, this project was the first to use repeated sampling of migratory tree-roosting bats over time, and the lessons learned here (e.g., parameter space should be adequately explored, and the samples and loci should be thoroughly vetted prior to drawing conclusions [76]) will be used to inform future monitoring efforts.

## 4. Recommendations

No single method can provide an estimate of Nc for hoary bats or any other migratory tree-roosting species. Obtaining basic ecological data, including demographic data and growth rates, for these species remains elusive. In the absence of estimates for Nc, a weight of evidence is needed to understand the viability of a species. Multiple methods, even if indirect, are necessary to assess the status and trends of these bat populations [41]. Results from acoustic and genetic sampling can provide estimates to inform population status and assess trends to enlighten stakeholders on whether mitigation measures are necessary to sustain a species.

Long-term, systematic data collection remains the most reasonable approach for reducing uncertainty for migratory tree-roosting bat populations. Statistically robust methods for acoustic monitoring, such as NABat, and estimating Ne at repeated intervals to examine evidence for genetic bottlenecks will elucidate species trends. Short-term investments that can support long-term success include altering the way in which bat acoustic surveys are conducted at wind energy facilities, contributing acoustic data to the NABat database, and collecting and analyzing genetic data from recovered carcasses.

### 4.1. Acoustic Monitoring Recommendations

In North America, standard preconstruction acoustic surveys for bats involve deploying acoustic monitoring stations at ground level and, when possible, on meteorological towers. The U.S. Fish and Wildlife Service (U.S. FWS) provides some guidance on the number of detectors and duration for these surveys [79]. Standard preconstruction acoustic surveys provide useful information on bat activity patterns and species occurrence at a proposed project. These data can also contribute to status and trends analyses if they are submitted to a robust relational and accessible database like NABat. Despite years of preconstruction surveys being conducted in association with wind energy development, little data exist in the NABat database from these studies at wind energy facilities [25], nor are they readily discoverable through another resource. Although these data are not equivalent to those collected following a common survey protocol and they do not represent a random sample, they can supplement a probabilistic sample to support occupancy analyses and contribute to an improved understanding of seasonal relationships and the full annual cycle for migratory bat populations [80]. Moreover, submitting these data to a common database can prevent data loss and support compliance with mandates for data transparency and sharing as imposed by agencies such as the New York State Energy Research and Development Authority [81]. Storing and collating these data in a common and accessible database can also make them more readily recoverable to permitting authorities or agencies that may desire to recompile data across space and time.

**Figure 3:**
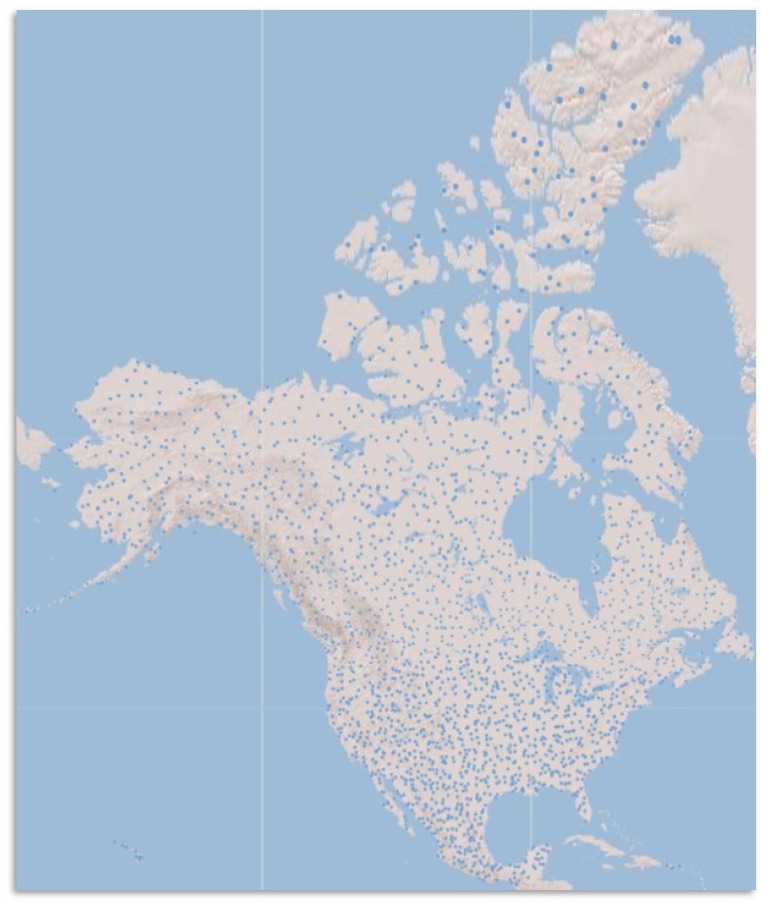
The top 1% priority grid cells from the NABat sampling frame are represented in blue and total 2275 cells across North America. Map is adapted from the North American Bat Monitoring Program Partner Portal [26].

A primary objective for standard preconstruction acoustic surveys was to determine whether preconstruction activity predicts postconstruction mortality. Because no reliable relationship has been established between these variables [82,83], it may no longer be beneficial to continue allocating resources for standard preconstruction acoustic monitoring. Instead, refocusing resources and efforts on NABat field methods and data submission protocols will enable broad-scale inferences about bat populations. These analyses and products can then support answering key preconstruction site evaluation questions for bats such as those posed in Tier 1 and Tier 2 of the U.S. FWS Land-Based Wind Energy Guidelines [79]. To serve this goal, it is necessary to sample high-priority cells (Figure 3) rather than any given wind energy facility because a random sample is what enables drawing inferences to unsampled locations. On the other hand, avoiding sampling in high priority cells that intersect with wind energy facilities, thereby resulting in the absence of these data in the full continental dataset, would present a source of bias that should be avoided.

There are instances where following the probabilistic sample is not feasible, such as conducting presence/absence surveys to fulfill obligations under Section 7 of the U.S. Endangered Species Act (16 U.S. Code § 1536). The Range-Wide Indiana Bat Survey Guidelines [84], for example, provides different requirements than the NABat protocol [23]. The former requires more sampling per unit area and is based on a set number of detector nights per area. Conversely, the NABat protocol is based on a set number of detector nights per site. These differences do not make these two protocols mutually exclusive, and with advance planning they can be aligned to meet multiple objectives.

**Figure 4:**
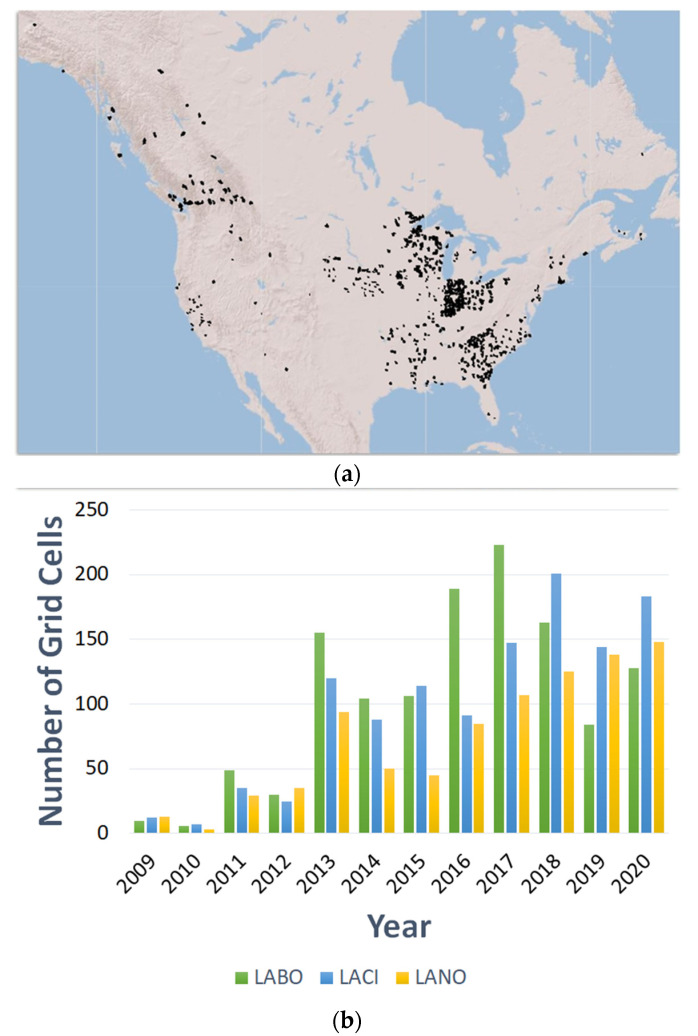
Mobile acoustic surveys have been conducted in more than 2000 grid cells across North America, represented in black (**a**), from 2009 through 2020 (**b**). From 2020 surveys, 128 cells contain data for eastern red bats (LABO), 179 cells contain data for hoary bats (LACI), and 145 cells contain data for silver-haired bats (LANO) [25]. Note: this is not a presence/absence map. Data and maps are adapted from the North American Bat Monitoring Program Partner Portal [26].

While stationary acoustic data are valuable in determining occupancy probability, mobile acoustic surveys serve as inputs to both occupancy and abundance models. In support of the objective to determine measures of abundance for migratory tree-roosting bats and to place wind turbine mortality in the context of population size and trends, it is recommended to adopt mobile acoustic surveys in high-priority cells. Although offshore transects are not currently part of the NABat protocol, these could be considered, and protocols developed if they are deemed feasible in the offshore environment. It may be possible to conduct boat surveys by adapting the methods used by Whitby et al. [51]. There are currently mobile acoustic data in the NABat database from more than 2000 grid cells across North America [25], but these data are not spatially balanced, and few mobile acoustic surveys are conducted in the west (Figure 4). Additional mobile transects in western states can supplement existing efforts.

Data collected in association with wind energy development can increase the quantity and quality of data available for analyses, offering in return data-driven updates to the probability of occupancy, species range maps, indices of abundance, and improved understanding of relationships with habitat features, available resources, and various stressors. To help achieve these goals, standard preconstruction bat data should be contributed to the NABat database, and where possible, transitions to mobile acoustic surveys following the NABat protocol are recommended.

### 4.2. Genetic Recommendations

#### 4.2.1. Comparative Studies

There is value in comparative genetic studies across species, geographic regions, and temporal scales. Continued genetic evaluation of opportunistically collected samples, such as those in Cornman et al. [76], with a further refinement of technical and analytic methods shows tremendous potential for genetic monitoring of migratory tree-roosting bat populations over space and time. We recommend a similar study design, with temporal replicates, be applied to eastern red and silver-haired bats to help answer the following questions: (1) do differences in estimates of genetic variation, population substructure (or lack thereof), and Ne correspond with previous genetic studies?; (2) what data are available regarding migratory habits, reproductive biology, etc.?; (3) do new observations meet expectations with respect to the ecology and life history of these species?; (4) if these new observations confirm expectations, what does the weight of evidence indicate about whether these populations are stable, decreasing, or increasing?; and (5) how can these data be used to inform mitigation strategies to better manage the species?

For contemporary samples, it will be important to locate sources of existing genetic material (e.g., tissue samples, DNA samples, DNA sequences, and genome data). It also will be necessary to identify partners willing to share tissue samples or carcasses from mortality searches at wind turbines during the next several years, with the goal of filling in data gaps in species or geographic coverage that may exist with the samples that are already available.

Future research can expand the geographic scope and temporal scale of hoary bat samples. It may be possible to use historical DNA samples to estimate the genetic parameters 10, 20, or even 100 years ago, which can be matched by geographic location to contemporary samples collected from wind energy facilities. The results of these analyses could be used to help answer the following questions: (1) is there evidence of a decline predating the development of wind energy?; (2) do changes in genetic diversity or Ne coincide with the expansion of wind energy development during the past 20–25 years? and (3) how do estimates of Ne and genetic diversity compare among different geographic regions that may be experiencing different stressors, such as wind energy development and climate change impacts?

Estimates obtained from population genetic studies will not provide direct estimates of Nc, but they can be used to monitor populations and population impacts over time and to make predictions about relative impacts to different species. Obtaining these estimates for hoary, eastern red, and silver-haired bats can reduce uncertainty regarding the species-specific impacts of wind energy development. It is crucial to invest resources to store and manage genetic samples from bats collected at wind energy facilities during the next decade. This collection should target two different scales that are reflected herein: (1) sample intensively at select geographic areas that have high levels of bat mortality for hoary, eastern red, and silver-haired bats; and (2) sample broadly across North America to capture the range-wide level of impacts for hoary, eastern red, and silver-haired bats. By dedicating collection efforts at these two scales, we will ensure sufficient sample sizes and that we have sampled the entire population for each species.

#### 4.2.2. Monitoring Studies

Taking multiple samples over time allows for monitoring genetic changes associated with various stressors [57]. This type of monitoring can be performed with microsatellites, mtDNA markers, and SNPs—with SNPs providing increased power to detect population subdivision if it exists, and increased precision in the resulting estimates of genetic parameters. For example, Bernger et al. [85] used microsatellites and mtDNA markers to determine that the low genetic variation currently observed in the kākāpō (*Strigops habroptilus*) resulted from a recent population bottleneck associated with European colonization. In another example, Cammen et al. [86] used RADseq to determine genetic signatures of historical bottlenecks in the contemporary genomes of both gray seals (*Halichoerus grypus atlantica*) and harbor seals (*Phoca vitulina vitulina*). These types of studies may also be informative for migratory tree-roosting bats.

We recommend sampling several wind energy facilities in each U.S. Fish and Wildlife Service region for continuous long-term monitoring (over 5, 10, and 20 years) for the three species of migratory tree-roosting bats with a focus on collecting tissue samples for subsequent genetic analyses. This could be achieved using a low-level monitoring effort, such as searching only gravel pads and roads at selected sites. The collection, processing, and storage of tissue samples and carcasses will require cooperation and coordination during standard postconstruction mortality monitoring efforts. The anticipated cost of tissue collection and storage are not anticipated to be a barrier to participation, but additional funding sources will be necessary to archive the samples and conduct the genetic analyses.

In addition to the strategic monitoring efforts described, we recommend that researchers continue to collect genetic samples from additional bat species that experience wind turbine mortality, especially as wind energy development expands into new areas. For example, Chipps et al. [87] examined patterns of genetic diversity in the southern yellow bat (*Lasiurus ega*) and the northern yellow bat (*Lasiurus intermedius*) using DNA from carcasses collected at wind energy facilities in south Texas, U.S. Yellow bats are in the same genus as hoary bats and eastern red bats, and also collide with wind turbine blades. Wind energy is relatively new in the region and represents a new source of mortality for these species. Chipps et al. [88] detected a significant female bias in collision mortality using molecular sex determination for the southern yellow bat. Estimates of historic Ne and genetic diversity in both yellow bat species were lower than those reported for the three migratory tree-roosting bats studied to date [87]. In addition to providing additional insights into the basic life history of these little-known species, this study (1) provided baseline genetic data that can be used in future genetic monitoring of yellow bats; and (2) demonstrated the value of using lower-cost methods, such as microsatellites and mtDNA sequence data, to investigate genetic variation in new species for which genomics tools are not yet available [54]. Furthermore, microsatellite analysis would be efficient for identifying siblings or other related pairs in carcass pools from wind energy facilities. If closely related individuals are consistently detected in random samples of migratory tree-roosting bats, this could be viewed as a potential indicator of severe population contraction.

Finally, we recommend the use of readily available and low-cost molecular methods to determine sex [88,89,90] and ascertain correct species identification using DNA barcoding [91] for bat carcasses salvaged at wind energy facilities. Several recent studies of capture, acoustic, and specimen records have documented range expansions in red and yellow bats [88,92,93,94,95], evening bats (*Nycticeius humeralis*) [96], Brazilian free-tailed bats (*Tadarida brasiliensis)* [97,98], and Seminole bats (*Lasiurus seminolus*) [99]. Clearly, more research is needed to fully understand the distribution of bats in North America, especially considering ongoing land use modification and climate change. Using these genetic tools will reduce uncertainty regarding the impacts of wind energy development on bats and allow for the development of effective sex- and species-specific management strategies.

## 5. Conclusions

While migratory tree-roosting bats are experiencing a relatively new source of mortality associated with wind energy development, they are simultaneously experiencing other persistent and emerging stressors, such as climate change [3,100], global declines in insects [101,102]—which are the primary food source for these bats, and continued habitat loss and degradation [3]. Given the anticipated expansion of wind energy development in the near term, it is imperative to prioritize gathering new data to help ascertain population status and trends. Building a weight of evidence, through the long-term, systematic collection of acoustic and genetic data, may improve our understanding of the population viability of migratory tree-roosting bats. These data can be leveraged to infer the impact of stressors on a species and subsequently determine whether mitigation is necessary to sustain populations. While an improved understanding has relevant applications beyond wind energy development, these efforts will directly benefit the wind energy–wildlife community by: (1) showing whether the population status is stable or decreasing, which may lessen the need for costly mitigation measures to reduce mortality associated with wind turbines; or (2) providing the data necessary to catalyze proactive and relatively modest mitigation measures in the short term to prevent more intensive and expensive measures that may be necessary if action is delayed.
